# Highly Productive Synthesis, Characterization, and Fluorescence and Heavy Metal Ion Adsorption Properties of Poly(2,5-dimercapto-1,3,4-thiadiazole) Nanosheets

**DOI:** 10.3390/polym10010024

**Published:** 2017-12-25

**Authors:** Chao Li, Shaojun Huang, Chungang Min, Ping Du, Yi Xia, Chaofen Yang, Qiuling Huang

**Affiliations:** 1 Research Center for Analysis and Measurement, Kunming University of Science and Technology, Kunming 650093, China; lichao2527@yeah.net (C.L.); minchungang@163.com (C.M.); dupin515@163.com (P.D.); xiayi0125@163.com (Y.X.); yangmlh@163.com (C.Y.); hql1975@eyou.com (Q.H.); 2 School of Materials Science and Engineering, Kunming University of Science and Technology, Kunming 650093, China

**Keywords:** poly(2,5-dimercapto-1,3,4-thiadiazole), synthesis, characterization, nanosheets, fluorescence, adsorption, heavy metal ion, mercury, properties

## Abstract

Poly(2,5-dimercapto-1,3,4-thiadiazole) (PBT) nanosheets were synthesized by chemical oxidative synthesis under mild conditions. The media, oxidant species, monomer concentrations, oxidant/monomer molar ratio, and temperature were optimized to achieve higher yields and better performance. The molecular structure, morphology, and properties of the nanosheets were analyzed by Fourier transform infrared (FT-IR), ultraviolet-visible (UV-Vis), and fluorescence spectroscopies, wide-angle X-ray diffraction (WAXD), matrix-assisted laser desorption/ionization/time-of-flight (MALDI-TOF) mass spectrometry, X-ray photoelectron spectroscopy (XPS), scanning electronic microscopy (SEM), transmission electron microscopy (TEM), and simultaneous thermogravimetry and differential scanning calorimetry (TG-DSC). It was found that the polymerization of 2,5-dimercapto-1,3,4-thiadiazole occurs via dehydrogenation coupling between two mercapto groups to form the –S–S– bond. PBTs show the highest polymerization yield of up to 98.47% and form uniform nanosheets with a thickness of 89~367 nm. poly(2,5-dimercapto-1,3,4-thiadiazole) polymers (PBTs) exhibit good chemical resistance, high thermostability, interesting blue-light emitting fluorescence, and wonderful heavy metal ion adsorption properties. Particularly, the PBT nanosheets having a unique synergic combination of three kinds of active –S–, –SH, and =N– groups with a moderate specific area of 15.85 m^2^ g^−1^ exhibit an ultra-rapid initial adsorption rate of 10,653 mg g^−1^ h^−1^ and an ultrahigh adsorption capacity of up to 680.01 mg g^−1^ for mercury ion, becoming ultrafast chelate nanosorbents with a high adsorption capacity. With these impressive properties, PBT nanosheets are very promising materials in the fields of water treatment, sensors, and electrodes.

## 1. Introduction

Over the past two decades, heterocyclic aromatic polymers have attracted a great deal of attention from both academic and industrial communities. Traditional heterocyclic aromatic polymers, such as polypyrrole and polythiophene, have been synthesized generally by either the electrochemical or chemical oxidative polymerization method [1,2,3]. In general, the electrochemical polymerization method allows for the fabrication of a uniform film with controllable thickness on the surface of electrodes. However, the low production caused by the small electrode area, the requirement of conducting substrates, the impurity of the resulting polymer, and pollution from the waste electrolyte restrict its wide applications [4]. By contrast, the chemical oxidative polymerization method shows some advantages involving mass production, controllable micro-morphology of polymer, easily adjustable reaction conditions, and simple doping-dedoping chemistry [5,6,7]. 

2,5-dimercapto-1,3,4-thiadiazole (bismuthiol, BT) is a heterocyclic aromatic mercaptan, which combines the features of pyrrole and thiophene according to its chemical structure. It has been reported that BT and its derivatives demonstrate excellent antibacterial properties [8,9,10,11,12] and strong coordination abilities to heavy metal ions [13,14,15] because of the sulfur (S)- and nitrogen (N)-rich subgroups and their extremely high total molar content (56%) in BT molecules. Hence, BT can be used to easily coordinate a variety of heavy metal ions, such as Co(II), Ni(II), Cu(II), Zn(II), and Au(III), to form BT-M^n+^ (2:1) metal coordination polymer [16,17], depending on the strong coordination ability of electron-rich nitrogen and sulfur subgroups.

Taking into account the chemical and electronic structures of 2,5-dimercapto-1,3,4-thiadiazole, one may find that the 2,5-dimercapto substituents in the monomer molecules are active points and the corresponding polymer can be produced by the electrochemical or chemical oxidative polymerization method. The content of functional imino and mercapto groups in the polymer molecules should greatly increase compared with that in the monomer molecules, thus it is reasonable to infer that the heavy metals ion adsorption and antibacterial abilities of 2,5-dimercapto-1,3,4-thiadiazole polymer will also be greatly enhanced. Accordingly, it is desirable to synthesize poly(2,5-dimercapto-1,3,4-thiadiazole) (PBT) as a promising adsorbent for heavy metal ions. However, the chemical oxidative polymerization of the 1,3,4-thiadiazole ring containing derivatives has been demonstrated to be very difficult, though the electrochemical polymerization method has proved to be feasible [18,19]. Sun, Qin, and coworkers [20,21,22] have attempted to synthesize a 1,3,4-thiadiazole ring containing polymers by using the chemical oxidative method, but merely obtained metal coordination polymers instead of genuine polymers like polypyrrole and polythiophene. The main difficulty in synthesizing a 1,3,4-thiadiazole ring containing polymers via chemical oxidative polymerization is as follows. The five-membered heterocyclic monomers generally contain 1–3 sulfur atoms and 2–3 nitrogen atoms and the synthetic condition, especially the selection of oxidant, is quite strict. Neither too strong nor too weak oxidants are favorable for their polymerizations, otherwise leading to a ring-opening reaction, peroxidation, or failure of initiation of the polymerization [23]. Moreover, the use of transition metal salts as oxidants readily leads to the generation of complexes or coordination polymers as mentioned above. To the best of our knowledge, the intensive research on the chemical oxidative synthesis of PBT and its performance has not been reported until now.

On the other hand, the discharge of sewage containing heavy metal ions, especially mercury ions and lead ions, can do irreversible harm to the environment and human beings [24,25,26]. If they are absorbed and enter into the human body, they will accumulate in the human body. For instance, mercury ions can cause nervous systemic poisoning, whereas lead ions can cause damage to vital organs, both leading to serious lesions and even inducing cancer [27]. Therefore, both the detection of heavy metal ions in the environment and the treatment of heavy metal ions pollution are essential. Generally, the adsorption method is considered as a traditional and popular treatment method for heavy metal ions pollution. The adsorption of heavy metals is mainly achieved by the porous structures of the adsorbent itself or the chemical groups [28,29]. PBTs are promising adsorbents for Hg and Pb because of the multi-functional groups on their molecular chains.

In this work, PBT polymers were productively synthesized by using a chemical oxidative polymerization of BT monomers. The effects of synthetic parameters including reaction medium, oxidant species, monomer concentration, oxidant dosage, and reaction temperature on the yields of PBTs were investigated and optimized. The chemical structures, micro-morphologies, and properties were studied by ultraviolet-visible (UV-Vis) spectroscopy, Fourier transform infrared (FT-IR) spectroscopy, matrix-assisted laser desorption/ionization/time-of-flight (MALDI/TOF) mass spectroscopy, X-ray photoelectron spectroscopy (XPS), wide-angle X-ray diffraction (WAXD), scanning electronic microscopy (SEM), transmission electron microscopy (TEM), fluorescence spectroscopy (FLS), and thermogravimetric analysis (TGA), respectively. Finally, the adsorption performance for heavy metal ions of the polymer was studied in detail.

## 2. Experimental Section

### 2.1. Chemicals

Ammonium persulfate [(NH_4_)_2_S_2_O_8_], 2,5-dimercapto-1,3,4-thiadiazole (bismuthiol, BT), iodine (I_2_), sodium hypochlorite (NaClO), hydrogen peroxide (H_2_O_2_), ferric chloride (FeCl_3_), perchloric acid (HClO_4_), *N*,*N*-dimethylformamide (DMF), *N*-methy1-2-pyrrolidone (NMP), tetrahydrofuran (THF), dimethyl sulfoxide (DMSO), acetone (CH_3_COCH_3_), nitromethane (CH_3_NO_2_), methanol (CH_3_OH), ethanol (C_2_H_5_OH), hydrochloric acid (HCl), ammonia (NH_3_·H_2_O), Hg(NO_3_)_2_, Pb(NO_3_)_2_, AgNO_3_, Co(NO_3_)_2_, Cr(NO_3_)_3_, Cd(NO_3_)_2_, Fe(NO_3_)_3_, Zn(NO_3_)_2_, Cu(NO_3_)_2_, Mg(NO_3_)_2_, NaNO_3_, KNO_3_, Al(NO_3_)_3_, dithranol (DIT), silver trifluoroacetate (CF_3_COOAg), and other chemicals were purchased from Chemical Reagent Corp. in Kunming, China. All reagents and solvents were of analytical reagent grade and used as received.

### 2.2. Synthesis of PBT Nanosheets

A typical synthesis of PBT was conducted as follows: 0.3005 g (2.0 mmol) of BT monomer was dissolved in 10 mL of absolute ethanol with the aid of stirring. The solution was transferred to a 100-mL glass-stoppered conical flask and stirred at 25 °C, followed by the dropwise addition of 30 mL of ethanol solution containing 0.7614 g (3.0 mmol) of I_2_. The solution exhibited a change in color from pale yellow to brown black once the oxidant solution was added, and a polymer precipitation appeared within 2–3 min. The reaction was carried out for an additional 24 h to ensure that all of the monomers were polymerized. At the end of the reaction, 200 mL of deionized water was added to the reaction mixture to adequately precipitate the polymer, and then the polymer precipitates were separated by centrifugation. The polymer precipitates were thereafter washed with excess deionized water and ethanol until the washing solution was colorless or light colored, and finally dried at 80 °C in air for 24 h, producing PBT nanosheet powders.

### 2.3. Fluorescent Property Investigation

First, 0.0010 g of PBT was dissolved in NMP to confect a 10 mg L^−1^ solution. This solution was stepwise diluted to 200, 150, 100, 50, and 10 μg L^−1^ with NMP. The excitation and emission spectra of these solutions were examined to find out which concentration could emit the strongest fluorescence. The optimum concentration was chosen for the subsequent experiments. On the other hand, the polymer organic solution was reacted with an aqueous solution of metal ion, and the resulting mixed solution was used to detect the fluorescence intensity change to exploit the potential of the polymer for the detection of certain metal ions. The typical fluorometric measurements of polymer-metal complex solutions were carried out as follows. Polymer-metal complex solutions used for fluorescence measurements were prepared by mixing 50 μL of polymer solution (10,000 μg L^−1^) in NMP with 1.0 mL of aqueous metal salt solutions (1.0 × 10^−4^ mol L^−1^) at room temperature, and then increasing the solution to a constant volume with the addition of 10.0 mL by NMP. The terminal concentrations of the polymer and metal ion were 50 μg L^−1^ and 1.0 × 10^−5^ mol L^−1^, respectively. The metal ions investigated were Na^+^, Mg^2+^, Al^3+^, K^+^, Cr^3+^, Fe^3+^, Co^2+^, Cu^2+^, Zn^2+^, Ag^+^, Cd^2+^, and Pb^2+^ as their nitrate salts. It was found that no precipitate was observed even though 20% (volume fraction) water was added into the polymer solution in NMP. All of the fluorescence measurements were operated right after the test solutions were prepared and thoroughly mixed. The fluorescence emission spectra were recorded based on these mixed solutions with a constant polymer concentration of 50 μg L^−1^. The slit widths for the excitation and emission were both fixed at 5.0 nm.

### 2.4. Adsorption Experiments

#### 2.4.1. Single Metal Ion Adsorption onto the Nanosheets

Different stock solutions containing one of the following metal ions, Pb^2+^, Hg^2+^, Ag^+^, Co^2+^, Cr^3+^, Cd^2+^, Fe^3+^, Zn^2+^ or Cu^2+^, were confected in advance. A typical adsorption operation taking Pb^2+^ solution as an example is described as follows. First, 50 mg of PBT nanosheets was added to 50 mL of Pb^2+^ solution at an initial concentration of 200 mg L^−1^, and the mixture was then vigorously stirred for 24 h in a water bath at 25 °C. After adsorption, the mixture was separated into a precipitate and a supernatant by centrifugation and the concentration of Pb^2+^ ions remaining in the supernatant was determined by inductively coupled plasma atomic emission spectrometry (ICP-AES).

The adsorption capacity (*Q*) and adsorption ratio (*q*) were calculated according to the following equations:

(1) $$Q=\frac{(C_{0}-C)V}{m}$$

(2) $$q=\frac{\left(C_{0}-C\right)}{C_{0}}\times 100\%$$

where *Q* (mg g^−1^) and *q* (%) are the adsorption capacity and adsorption ratio, respectively; *C*_0_ (mg L^−1^) and *C* (mg L^−1^) are the initial concentration of the metal ion and the metal ion concentration after adsorption, respectively; *V* (L) is the initial volume of metal ion solution; and *m* (g) is the weight of the nanosheets added.

#### 2.4.2. Adsorption Selectivity

The mixed cation solution containing Pb^2+^, Hg^2+^, Ag^+^, Co^2+^, Cr^3+^, Cd^2+^, Fe^3+^, Zn^2+^, and Cu^2+^ ions were used to examine the adsorption selectivity of PBT nanosheets. All of the metal ions used were in the same concentration of 20 mg L^−1^. In the examination, 50 mg of PBT nanosheets was added into 50 mL of mixed cation solution. The mixture was then vigorously stirred at 25 °C for 24 h. After adsorption, the mixture was separated into a precipitate and a supernatant by centrifugation for further analyses. The adsorption capacity and adsorption ratio of each metal ion in the mixed cation solution were calculated using the method described in Section 2.4.1.

### 2.5. Characterization and Measurements

The UV-Vis spectra of the polymer solutions or dispersions (500 mg L^−1^) in NMP were recorded on a TU-1901 UV-Vis spectrophotometer (Beijing Purkinje General Instrument Co., Ltd., Beijing, China) in a wavelength range of 190–900 nm at a scanning rate of 400 nm min^−1^. Fourier transform infrared (FT-IR) spectra of the PBT/KBr (weight ratio of 1:60) pellets were recorded on a Bruker TENSOR 27 FT-IR spectrometer (Karlsruhe, Germany) with a resolution of 4 cm^−1^, by the transmittance method. Wide-angle X-ray diffraction (WAXD) scanning was carried out on a Rigaku International Corporation D/max 2000 X-ray diffractometer (Tokyo, Japan) with Cu Kα radiation in a Bragg angle range of 10–80° at a scanning rate of 1° min^−1^. The matrix-assisted laser desorption/ionization/time-of-flight (MALDI/TOF) mass spectra of PBT in THF, using dithranol (DIT) as a matrix and silver trifluoroacetate (CF_3_COOAg) as a sensitizer, were recorded on a Bruker Daltonik GmbH autoflex speed TOF/TOF mass spectrometer (Bruker Corporation, Billerica, MA, USA). The XPS spectra were taken by a PHI5000 Versaprobe-II multifunctional scanning and imaging photoelectron spectrometer (Physical Electronics Inc., Chigasaki, Japan) equipped with an Al Kα X-ray source. Thermogravimetric (TG) analysis was performed on a NETZSCH STA 449F3 TG-DSC simultaneous thermal analyzer (NETZSCH Group, Bavaria, Germany) in a temperature range from room temperature to 1000 °C at a heating rate of 20 °C min^−1^ under a flow of nitrogen. The fluorescence excitation and emission spectra were obtained by a F-7000 fluorescence-phosphorescence spectrophotometer (Hitachi High-Technologies Corporation, Tokyo, Japan). The size and morphology of the PBT nanosheets were analyzed by a FEI Tecnai G2 TF30 S-Twin field emission TEM (Eindhoven, The Netherlands) and a FEI Quanta X50 scanning electron microscope (SEM). The samples for TEM observation were prepared by dropping their suspension in alcohol onto copper mesh, while the samples for SEM observation were prepared by dropping their suspension in alcohol onto conductive tape. The atomic emission spectra of metal ions in water samples were recorded on a Prodigy High Dispersion ICP–AES (Leeman Labs Inc., Hudson, NH, USA). Nitrogen adsorption/desorption measurements at 77.4 K were performed after degassing the PACA powders under high vacuum at 100 °C for at least 20 h using a DZF-6030A vacuum drying oven (Suzhou Jiangdong Precision Instruments Co., Ltd., Suzhou, China). The specific surface area was calculated by applying the Brunauer–Emmett–Teller (BET) model to the adsorption or desorption branches of the isotherms (N_2_ at 77.4 K).

## 3. Results and Discussion

### 3.1. Synthesis PBT Nanosheets 

#### 3.1.1. Selection of Polymerization Media

**Figure 1:**
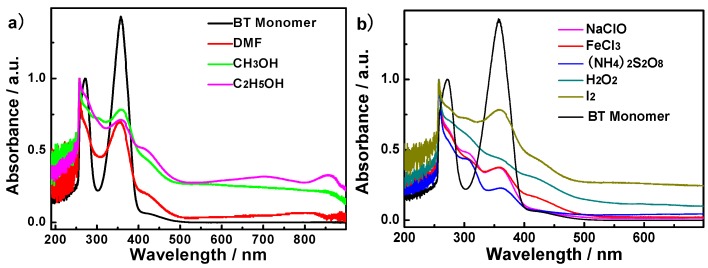
The UV-Vis spectra of 2,5-dimercapto-1,3,4-thiadiazole (BT) monomer and poly(2,5-dimercapto-1,3,4-thiadiazole) (PBT) polymers synthesized (**a**) in different media with an I_2_/BT molar ratio of 1.5, and (**b**) with the oxidant/BT molar ratio of 1.5 in ethanol, together with an initial BT concentration of 50 mmol L^−1^ at 25 °C for 24 h.

The screening of an appropriate medium is very important for chemical oxidative polymerization [30,31]. The solubility tests show that BT can be dissolved well in DMF, CH_3_OH, C_2_H_5_OH, NMP, DMSO, CH_3_COCH_3_, THF, and 1 mol L^−1^ NH_3_·H_2_O, but partially dissolved in CH_3_NO_2_ and slightly dissolved in 1 mol L^−1^ HCl, as summarized in Table S1. Among these media, DMF, CH_3_OH, and C_2_H_5_OH can also dissolve commonly used oxidants such as (NH_4_)_2_S_2_O_8_, FeCl_3_, and I_2_, so these three media can be used for the polymerization of BT. However, weak solvents as CH_3_NO_2_ and 1 mol L^−1^ HCl, strong volatile THF and CH_3_COCH_3_, and alkaline NH_3_·H_2_O, together with high boiling solvents like NMP and DMSO are not suitable for the polymerization of BT monomers. Upon adding the I_2_ oxidant solution to the BT monomer solution, the color of the monomer solution changed immediately from transparent pale yellow to brown yellow and then to brown black, and yellow precipitates appeared at the bottom of the reactor within several minutes, indicating that the chemical oxidative polymerization of BT monomers really occurred. When I_2_ acted as the oxidant and CH_3_OH, C_2_H_5_OH, or DMF was used as the oxidant, the yields of 82.23%, 80.20%, and 38.5% were obtained, respectively. As can be seen in Figure 1a, the UV-Vis absorption at a long wavelength (700–900 nm) was relatively stronger when C_2_H_5_OH was used, indicating the higher average molecular weight of the polymer. On the other hand, since C_2_H_5_OH is also an environmentally friendly reaction medium, we choose ethanol as the best polymerization medium in the subsequent synthesis.

#### 3.1.2. Screening of the Oxidant Species

**Scheme 1:**
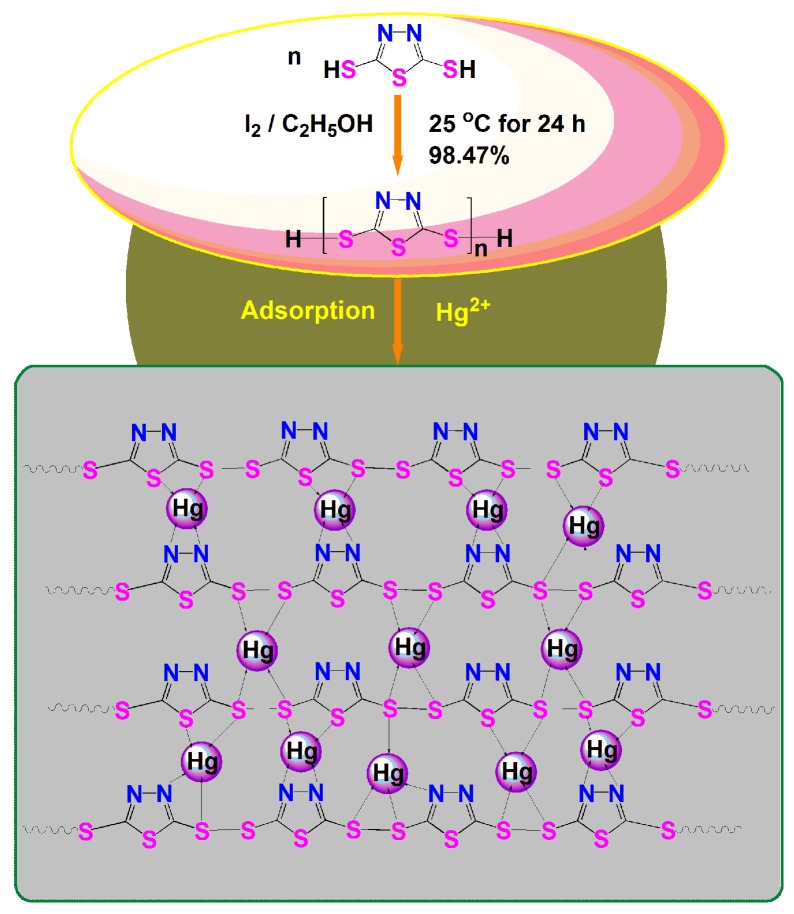
Chemical oxidative polymerization of 2,5-dimercapto-1,3,4-thiadiazole (BT) and the proposed adsorption mechanism of PBT nanosheets for Hg^2+^.

The selection of the oxidant is also critical for the chemical oxidative polymerization of BT monomers (Scheme 1), which is related to the success or failure of the initiation of the polymerization. A variety of oxidants, including I_2_, H_2_O_2_, (NH_4_)_2_S_2_O_8_, FeCl_3_, NaClO, and HClO_4_, were explored to polymerize BT monomers, and the resulting yields were 82%, 91.80%, 74.71%, 100.32%, 18.08%, and 0%, respectively. When FeCl_3_ was used, the yield exceeded 100%, because iron ions are easy to complex with the S and N subgroups in the polymer chains and cannot be totally removed. When HClO_4_ was used, no product was obtained at all. It should be noted that the polymerization of BT can produce hydrogen ions, but too many hydrogen ions may inhibit the polymerization reaction and promote its reverse reaction. In general, HClO_4_ has been used as a strong acid instead of an efficient oxidant in the polymerization of heterocyclic aromatic monomers. Small amounts of oligomers might be produced when perchloric acid was used as an oxidant, but these low molecular weight oligomers were difficult to be precipitated from the reaction solution and were easily washed off during the purification process. By contrast, when I_2_ or H_2_O_2_ was used as the oxidant, the yield was considerable, and the final reduced products of the oxidants were I^−^ and H_2_O, respectively, which greatly facilitates post-processing so as to obtain a pure product. From the UV-Vis spectra in Figure 1b, one can see that the PBTs prepared with I_2_ and H_2_O_2_ exhibited stronger absorptions at 358 nm and longer wavelengths when compared with those prepared with other oxidants. Thus, I_2_ and H_2_O_2_ were selected as the optimal oxidants for polymerizing BT monomers.

#### 3.1.3. Optimization of the BT Concentrations, Oxidant/BT Molar Ratios, and Polymerization Temperature

In the oxidation system of I_2_/C_2_H_5_OH, when the molar ratios of oxidant to monomer were changed from 0.25 to 0.5, 1, 1.5, and 2, the polymerization yields reached 8.72%, 68.56%, 80.20%, 98.47%, and 96.45%, respectively. It can be seen that the polymerization yield increased rapidly and then reached the maximum with increasing the I_2_/BT molar ratios from 0.25 to 1.5. Further increasing the I_2_/BT molar ratio barely changed the polymerization yield. As the I_2_/BT molar ratio was fixed at 1.5 and the BT concentration increased from 30.65 to 43.75, and 50 mmol L^−1^, the yield increased dramatically from 58.72% to 65.40% and 98.47%, and then decreased slightly (92.91%) upon further increasing the BT concentration to 58.25 mmol L^−1^. Obviously, few reaction active centers are created at low monomer concentrations and low I_2_/BT molar ratios, whilst the growth of polymeric chains is easier to be terminated with very high monomer concentrations and very high I_2_/BT molar ratios [32,33], explaining why a good polymerization yield (98.47%) of PBT was obtained at a moderate I_2_/BT molar ratio (1.5) and a moderate BT concentration (50 mmol L^−1^). With raising the polymerization temperatures from 0 °C to 5 °C, 25 °C, 40 °C, and 50 °C, the yields first rose and then fell, and they were 31.17%, 72.17%, 98.47%, 77.85%, and 56.18%, respectively. The PBTs exhibited maximal polymerization yields at 25 °C. Too high a temperature, however, induces drastic chain termination, whereas too low a temperature can cause a low speed of polymerization and less monomer initiation [32,33]. This explains why a moderate temperature of 25 °C is the optimal temperature for the synthesis of PBT.

In the oxidation system of H_2_O_2_/C_2_H_5_OH, the H_2_O_2_/BT molar ratios, monomer concentration, and polymerization temperature were optimized according to the yields in the same way. It was found that the highest yield of up to 97.20% was obtained when the PBT was synthesized with an initial BT concentration of 58.25 mmol L^−1^ and an H_2_O_2_/BT molar ratio of 3.53 in ethanol at 25 °C for 24 h. In fact, the PBT polymer synthesized in the I_2_/C_2_H_5_OH oxidation system has a nearly equal yield compared with that synthesized in the H_2_O_2_/C_2_H_5_OH oxidation system.

### 3.2. Chemical Structure of PBT Nanosheets

#### 3.2.1. FT-IR Spectra

**Figure 2:**
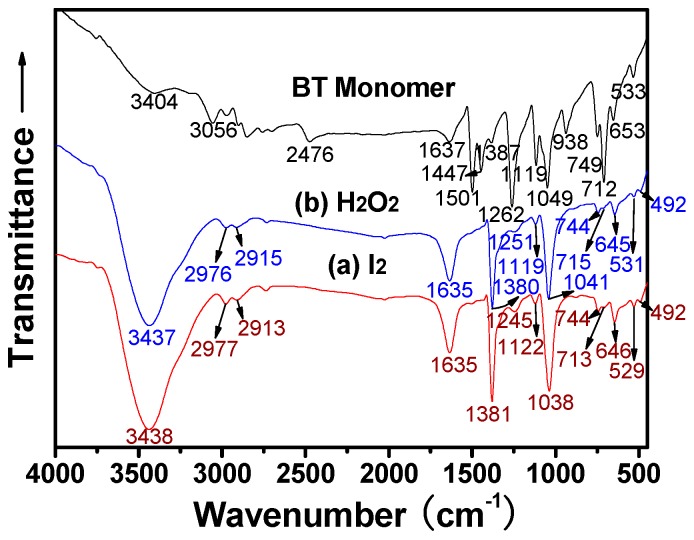
FT-IR spectra of BT monomer and PBT polymers prepared with (a) an I_2_/BT molar ratio of 1.5 and an initial BT concentration of 50 mmol L^−1^, and (b) an H_2_O_2_/BT molar ratio of 3.53 and an initial BT concentration of 58.25 mmol L^−1^ in ethanol at 25 °C for 24 h.

Figure 2 shows the FT-IR absorptions of BT monomer and PBT polymers synthesized with I_2_ and H_2_O_2_. For BT monomer, the characteristic bands appearing at 2476, 1637, 1501, 1387, 1262, 1119, 1049, 938, 749, 712, 653, and 533 cm^−1^ can be assigned to –S–H stretch, C=N stretch, N–H in-plane deformation, thiadiazole ring skeleton stretch, thioamide II mode, thiadiazole ring skeleton stretch, N–N stretch, C=S stretch, N–H torsion, C–S–C endocyclic asymmetric stretch, C–S–C endocyclic symmetric stretch, and S–C–S stretch (Table S4) [8,34,35,36]. However, compared with BT monomer, it was observed that the –S–H stretch band at 2476 cm^−1^ disappeared and a new band at 492 cm^−1^, corresponding to S–S stretch, appeared in each PBT polymer spectrum, which signifies that the polymerization of BT monomer occurs through –S–S– bond linkages [37,38]. For PBT polymers, the bands at 1501, 1262, 938, 749, and 712 cm^−1^ weakened and shifted slightly in position or disappeared because the Fermi resonance modes of the monomer units were basically inhibited after the formation of macromolecular chains [8]. On the other hand, the bands at 1637, 1387, and 1049 cm^−1^ in the BT monomer spectrum were shifted slightly to lower wavenumbers and became obviously more intense after BT monomers were polymerized because that the formation of macromolecular chains changed the electron cloud density of each group. Interestingly, the PBT polymers synthesized with I_2_ and H_2_O_2_ were similar in frequency and morphology of FT-IR absorptions, indicating their similar macromolecular structures. 

#### 3.2.2. UV-Vis Spectra

Figure S1 shows the UV-Vis absorption spectra of the NMP solution of BT monomer and PBT polymers. BT monomer exhibited two strong absorption peaks centered at 272 and 358 nm, which are assigned to the π→π* transition of the thiadiazole moiety and the n→π* transition of the –S–(HS)C=N– moiety in a BT molecule [39,40]. However, the spectrum of PBT polymer synthesized with H_2_O_2_ presented a broad and tailed absorption peak at 270 nm, which may be relative to the formation of polymer macromolecular structures. By contrast, the spectrum of PBT polymer synthesized with I_2_ showed four peaks centered at 258, 358, 702, and 855 nm, respectively. These two new absorptions at 702 and 855 nm are both attributed to an intramolecular charge transfer of the entire macromolecules [41]. The slight blue shift of the first peak from 272 nm to 258 nm after polymerization may be also ascribed to the formation of polymer macromolecules [39]. In summary, the differences in UV absorptions of the polymers and monomer demonstrated that the polymerization of BT monomers indeed happened. Additionally, the results indicate that the PBT polymer synthesized with I_2_ may possess a higher average molecular weight as compared with that synthesized with H_2_O_2_. 

#### 3.2.3. Wide Angle X-ray Diffraction

**Figure 3:**
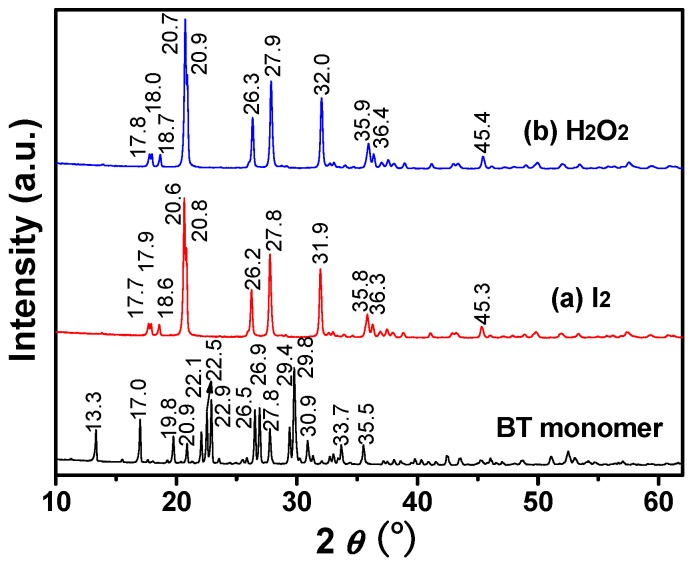
Wide-angle X-ray diffraction (WAXD) diffractograms of BT monomer and PBT polymers prepared with (a) an I_2_/BT molar ratio of 1.5 and an initial BT concentration of 50 mmol L^−1^, and (b) an H_2_O_2_/BT molar ratio of 3.53 and an initial BT concentration of 58.25 mmol L^−1^ in ethanol at 25 °C for 24 h.

The wide-angle X-ray diffraction (WAXD) spectra of PBT polymer powders and BT monomer are shown in Figure 3. Both the polymers and the monomer presented multiple sharp diffraction peaks, but both the number of diffraction peaks and peak positions were not similar (See Table S5 and Figure 3). The PBT polymers synthesized with I_2_ and H_2_O_2_ exhibited the same number of diffraction peaks, whilst the 2*θ* values of these diffraction peaks varied by about 0.1° (See Table S5). The former displayed five main diffraction peaks at 20.6°, 20.8°, 26.2°, 27.8°, and 31.9°, whereas the latter displayed five main diffraction peaks at 20.7°, 20.9°, 26.3°, 27.9°, and 32.0°. These results reveal the crystalline nature of the PBT polymers and a slight difference in crystal structures [17], supporting the SEM and TEM results. It was found that the number of diffraction peaks or their 2*θ* values of the polymer may change if the electrochemical or chemical polymerization method is varied. Our PBT polymers differ significantly in the number of diffraction peaks and 2*θ* values with the crystalline PBT chemically synthesized with ammonium persulfate in mixed solvent CH_3_OH/H_2_O and the electrochemically synthesized less crystalline PBT [42]. Compared with the PBT polymers, BT monomer showed more diffraction peaks, mainly at 13.3°, 17.0°, 19.8°, 20.9°, 22.1°, 22.5°, 22.9°, 26.5°, 26.9°, 27.8°, 29.4°, 29.8°, and 30.9°. The different peaks suggest changes in the unit-cell symmetry between the polymers and monomer [42]. 

#### 3.2.4. MALDI/TOF Mass Spectra

**Figure 4:**
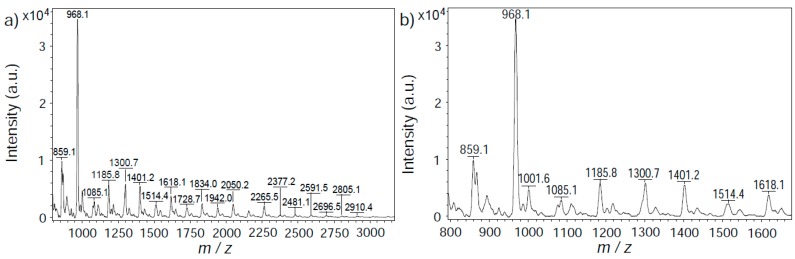

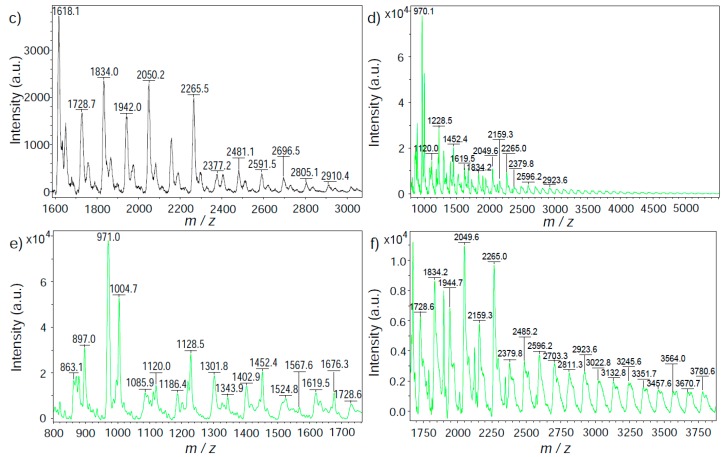
MALDI/TOF mass spectra of tetrahydrofuran (THF)-soluble part of PBT salts prepared with (**a**–**c**) an I_2_/BT molar ratio of 1.5 and an initial BT concentration of 50 mmol L^−1^, and (**d**–**f**) an H_2_O_2_/BT molar ratio of 3.53 and an initial BT concentration of 58.25 mmol L^−1^ in ethanol at 25 °C for 24 h.

The molecular weights of PBT polymers were estimated by MALDI/TOF mass spectrometry (Figure 4a–f) and the proposed compositions regarding each quasi-molecular ion are summarized in Table S6. The molecular weight, i.e., the degree of polymerization of PBTs, depended significantly on the oxidant species applied. It was found from Figure 4a–c that the molecular weights of the THF soluble part of PBT polymer salts prepared with I_2_ are mostly in the range of 859.1~2910.4, corresponding to the polymerization degree of 6~16, whilst those prepared with H_2_O_2_ are mainly in the range of 863.1~3780.6, corresponding to the polymerization degree of 6~24 (Figure 4d–f). It is surprisingly noted that the actual polymerization degree cannot be simply calculated as the *m*/*z* value divided by the mass of a BT monomer unit (–C_2_N_2_S_3_–, *m* = 148.21). The calculated mass differential (Δ*m*/*z*) between some neighboring quasi-molecular ions is about 105~112, a value approximately equivalent to the mass of Ag^+^ (107.9). This reveals that the PBT molecules were forming metal ion adducts with Ag^+^. Since the –S–, –SH, and =N– groups on the PBT molecules have a strong affinity toward Ag+, H^+^, K^+^, Na^+^, or other cations from the silver trifluoroacetate sensitizer, dithranol matrix, THF solvent, or glassware, the sulfur and nitrogen atoms are sometimes ionized by these ions in order to form quasi-molecule ions or metal ion adducts (See Table S6) [32,33]. The –SH terminal groups are sometimes eliminated from the polymer chains because of laser irradiation. Thus, the quasi-molecule ions or metal ion adducts can be attributed to a composition as expressed in the general Formula (3):

[H(C_2_N_2_S_3_)_n_H + aAg + bK + cNa + dH − eS]^+^ (n ≥ 2; a, b, c, d, e = 0, 1, 2, 3, …) (3)

As can be seen from Table S6 of the Supplementary Materials, the THF soluble parts of PBTs synthesized with different oxidants have wide polymer chain length distributions and similar chain structures but different polymerization degrees. Furthermore, the calculated values of *m*/*z* for the proposed compositions are in good agreement with the experimental values of *m*/*z*, suggesting the rationality of the proposed compositions as expressed in Formula (3). After all, about 85 wt % of PBT polymer salts are insoluble in THF, and their molecular weights can be measured neither by MALDI/TOF mass spectrometry nor by high temperature gel permeation chromatography (HT-GPC). Presumably, the insoluble parts may have far higher molecular weights than the soluble parts. On the other hand, PBT polymer prepared with I_2_ exhibited weaker solubility in common solvents including THF, NMP, DMSO, C_2_H_5_OH, CH_3_OH, CH_3_COCH_3_, and CH_3_NO_2_ in comparison with that prepared with H_2_O_2_, as can be seen from Table S1. This indicates that the PBT polymer prepared with I_2_ possesses relatively higher molecular weights. 

#### 3.2.5. XPS Analysis

**Figure 5:**
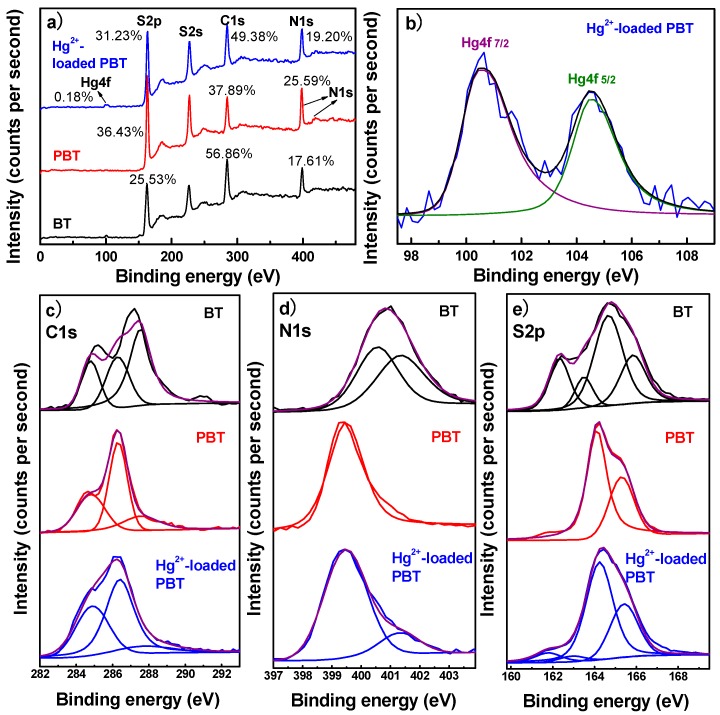
(**a**) XPS wide scan spectra of BT, PBT, and Hg^2+^-loaded PBT, and high-resolution XPS spectra of (**b**) Hg 4f, (**c**) C 1s, (**d**) N 1s, and (**e**) S 2p.

**Scheme 2:**
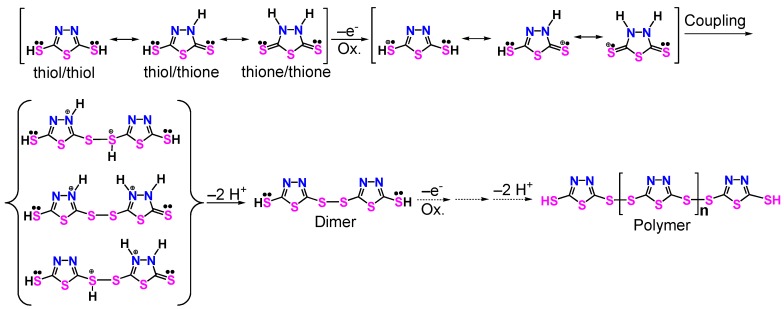
Proposed polymerization mechanism of BT.

XPS analysis was initially conducted for BT monomer and PBT polymer prior to adsorption in order to characterize the available functional groups and reveal the polymerization mechanism. Figure 5 shows XPS wide scan spectra of BT, PBT, and Hg^2+^-loaded PBT, as well as high-resolution XPS spectra of Hg 4f, C 1s, N 1s, and S 2p. The XPS binding energies (BEs) obtained from the Hg 4f, C 1s, N 1s, and S 2p core levels, in addition to their atomic concentrations and assignments, are listed in Table 1. All of the C 1s, N 1s, and S 2p regions were analyzed by peak deconvolution. The C 1s spectrum of BT monomer was deconvoluted into three component peaks at 284.8, 286.3, and 287.5 eV, and they were attributed to adventitious carbon (C–C/C=C), C–N/C=N, and C–S/C=S, respectively (Figure 5c) [43]. The C 1s XPS of PBT polymer, however, does not clearly show significant changes of BEs in comparison with that of BT monomer, indicating that the coupling reaction of BT units does not occur on the heterocyclic carbon. The N 1s region of BT monomer was deconvoluted into two component peaks at 400.5 and 401.3 eV and they were associated with –N=/N–N and N–H, respectively (Figure 5d) [4,44]. The absence of a BE at 401.3 eV for N–H and an apparent shift of the BE from 400.5 to 399.5 eV for –N=/N–N were observed in the N 1s XPS spectrum of PBT polymer. This could probably be explained by the inhibition of thiol/thione or thione/thione tautomerization [8] and an increase in electron cloud density of the nitrogen atom after polymerization (Scheme 2) [17,45,46]. The S 2p region of BT monomer was decomposed into two groups of component peaks at 162.3/163.5 eV and 164.7/165.8 eV, which are ascribed to S–H and heterocyclic sulfur (C–S and C=S), respectively. By contrast, as can be seen from the S 2p spectrum of PBT polymer (Figure 5e), the former group of peaks at 162.3 and 163.5 eV for S–H disappeared and the latter group of peaks at 164.7 and 165.8 eV shifted negatively by 0.5–0.6 eV. Further, the atomic concentration of heterocyclic sulfur (C–S and C=S) and S-S groups in PBT polymer increased by about 18.6% in comparison with that in BT monomer (see Table 1). All of these confirm that the coupling of BT monomers occurs via the S-S linkage, which basically agrees with the FT-IR spectral results.

### 3.3. Polymerization Mechanism of PBT

To gain a deeper insight into PBT structure and its polymerization mechanism, the proportion of frontier orbitals and the atomic electron spin density of BT monomer (Figure S2) were calculated at the B3LYP/6-31G (d) level using Gaussian 09 software (Tables S2 and S3). The results reveal that, in the case of BT monomer, the proportions of atoms in the highest occupied molecular orbital (HOMO) (Table S2) follow the order: S(2) = S(5) > N(3) = N(4) > C(2) = C(5) >> S(1). That is to say, S(2) and S(5) atoms are more negatively charged. Since the reaction between the active molecules takes place mostly on the frontier molecular orbitals and the neighboring orbitals, it can be inferred that the coupling polymerization of BT occurs preferentially at the S(2) and S(5) positions. Besides, electron spin density (ESD) is another important factor determining the possibility of oxidative polymerization coupling occurring [47,48]. The ESD of BT monomer radical cations was calculated at the B3LYP/6-31G (d) level, as listed in Table S3. The results imply that S(2) and S(5) atoms display the highest ESD, further signifying that S(2)–S(5) coupling occurs during chemical oxidative polymerization.

Based on FT-IR, MALDI/TOF mass, XPS spectra, and theoretical calculations, one could logically draw a conclusion that the chemical oxidative polymerization of BT occurred most likely through S(2)–S(5) coupling between 2- and 5-mercapto groups. This coincides well with the chemical oxidative polymerization coupling mechanism of pyrrole [49], thiophene [50], and 2-aminothiazole [40]. Therefore, we propose a polymerization mechanism of BT, as illustrated in Scheme 2. The initial step includes forming the radical cation through an electron transfer from the C–S or C=S moiety of BT tautomer to the oxidant. The next step is the reaction between the radical cations, thus forming the dication dimer, followed by a deprotonation to produce neutral dimers. The dimers can be oxidized more easily than the monomers and are thus able to form new biradical dications. Then, the as-formed biradical dication dimers would couple with either the radical cation monomers or themselves to achieve chain propagation, eventually leading to the formation of long PBT polymer chains. 

### 3.4. Morphological Characterization of Layered PBT Nanosheets

**Figure 6:**
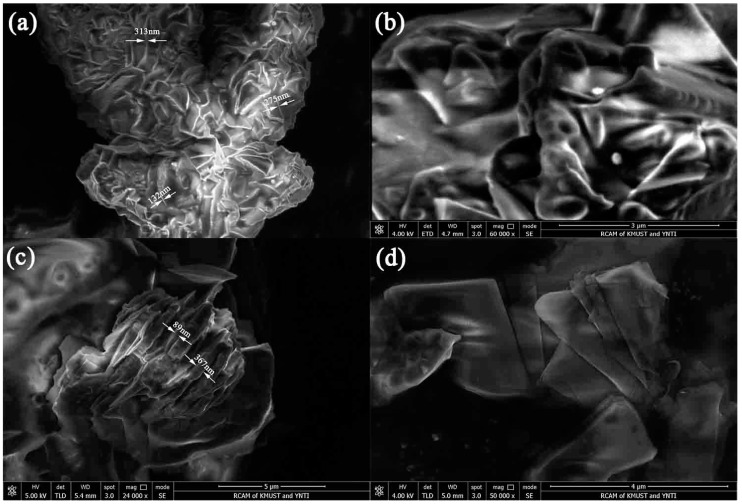
SEM images of PBT nanosheets synthesized with (**a**,**b**) an H_2_O_2_/BT molar ratio of 3.53 and an initial BT concentration of 58.25 mmol L^−1^, and (**c**,**d**) an I_2_/BT molar ratio of 1.5 and an initial BT concentration of 50 mmol L^−1^ in ethanol at 25 °C for 24 h.

**Figure 7:**
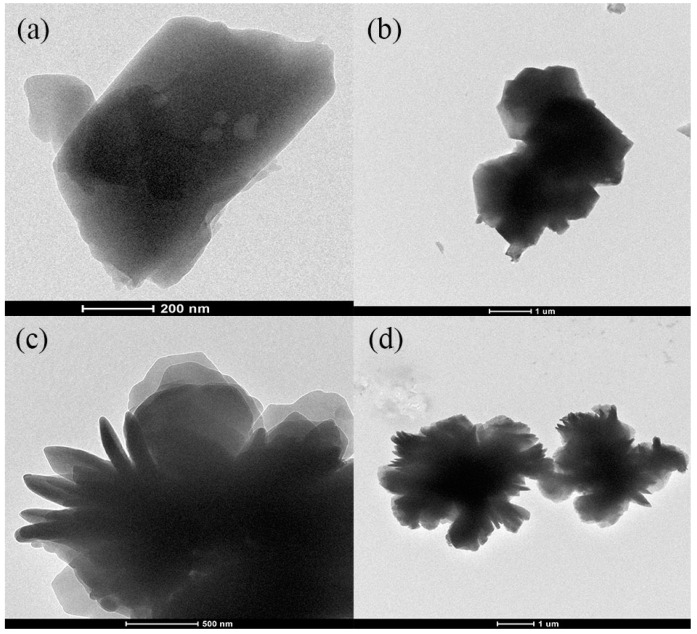
TEM images of PBT nanosheets synthesized with (**a**,**b**) an H_2_O_2_/BT molar ratio of 3.53 and an initial BT concentration of 58.25 mmol L^−1^, and (**c**,**d**) an I_2_/BT molar ratio of 1.5 and an initial BT concentration of 50 mmol L^−1^ in ethanol at 25 °C for 24 h.

Figure 6 and Figure 7 show the SEM and TEM images of layered PBT nanosheets made with H_2_O_2_ and I_2_. The as-synthesized PBT polymers display a uniform and compact layered sheet-like morphology. The thickness of the layered PBT nanosheets made with H_2_O_2_ and I_2_ was found to be in the range 132–313 nm and 89–367 nm, respectively, as shown in Figure 6. The layered morphologies of the synthesized PBTs are further characterized by TEM analysis, as depicted in Figure 7. The layered morphologies of PBT nanosheets are clearly visible, which is consistent with the results of SEM observation. There is no deformation of the morphology of layered PBT nanosheets under the high electron beam of TEM, suggesting the stability of the synthesized layered PBT nanosheets. The formation of these layered nanosheets is related to the excessive growth, i.e., secondary growth, of the polymer and π-π stacking interactions of the polymer molecular chains [51,52,53,54]. 

### 3.5. Properties of PBT Nanosheets

#### 3.5.1. Chemical Resistance

As summarized in Table S1, the PBT polymers demonstrate quite different chemical resistance as compared with BT monomer because the former have far higher molecular weights and thus much stronger interaction and cohesion between the macromolecular chains. The two PBT polymers are insoluble, slightly soluble, or at most partially soluble in THF, DMF, NMP, DMSO, C_2_H_5_OH, CH_3_OH, and CH_3_COCH_3_, but BT monomer is totally soluble in these seven solvents. The solvent resistance of the PBTs in 1 mol L^−1^ HCl and CH_3_NO_2_ is also better than that of BT monomer, because the PBTs are totally insoluble in these two solvents but BT monomer is only partially or slightly soluble in them. This good chemical resistance is one of the necessary properties of PBTs for applications as excellent adsorbents, discussed below. Certainly, the PBTs and BT monomer are both completely soluble in 0.1 mol L^−1^ NH_3_·H_2_O because there are so many weakly acidic mercapto groups in PBT polymers and BT monomer. 

#### 3.5.2. Thermostability

**Figure 8:**
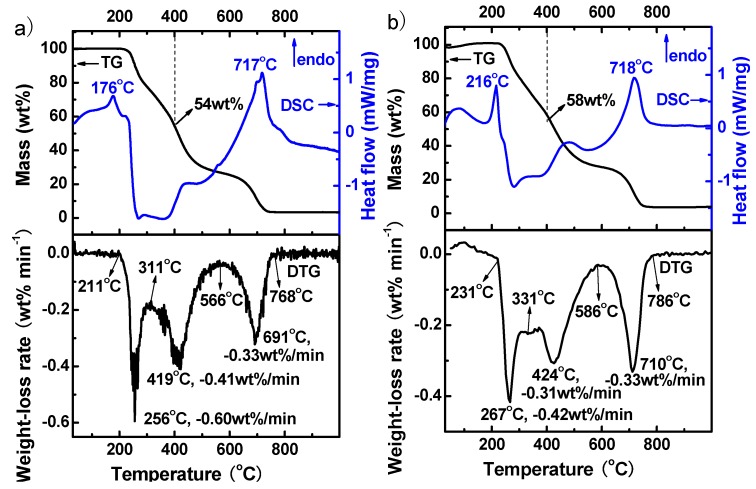
Thermogravimetry (TG), differential thermogravimetry (DTG), and differential scanning calorimetry (DSC) curves in nitrogen for PBT powder prepared with (**a**) I_2_ and (**b**) H_2_O_2_.

Thermal analysis of the PBT polymer powder was performed using thermal gravimetric analysis (Figure 8) under a N_2_ atmosphere by varying the heating temperature from 30 to 1000 °C at a heating rate of 20 °C min^−1^. The results show that PBT polymers have good thermostability. The PBT synthesized with I_2_ has no weight loss when the temperature is lower than 211 °C, and the weight loss is only 46 wt % at 400 °C. By contrast, the PBT synthesized with H_2_O_2_ has no weight loss when the temperature is lower than 231 °C, and the weight loss is only 42 wt % at 400 °C. However, BT monomer could be decomposed at its melting point (162 °C) and then lose all its weight. This implies that PBT polymers have a much higher thermal stability than BT monomer. The thermogravimetric (TG) and differential thermogravimetric (DTG) plots of PBT synthesized with I_2_ in Figure 8a exhibit three-stage thermal degradation at about 256, 419, and 691 °C, indicating that the polymer decomposition takes place in three steps. The first step (211–311 °C) with a *T*_DTG_ of 256 °C shows a weight loss of 24.4%, which should be caused by the exclusion of the residual dopant, the oxidant and its reduced product trapped in the samples, the end-mercapto groups, and a small part of oligomers [30]. The second step (311–566 °C) with a *T*_DTG_ of 419 °C exhibits a major loss of 48.7% owing to the gradual decomposition of the polymer backbones. The third step (566–768 °C) with a *T*_DTG_ of 691 °C presents a 23.5% weight loss due to the further pyrolysis of residual char formed after the initial PBT decomposition [55]. Similarly, the PBT synthesized with H_2_O_2_ also presents three-step decomposition at 267 °C (231–331 °C), 424 °C (331–586 °C), and 710 °C (586–786 °C) (Figure 8b), showing a weight loss of 26.3%, 45.8%, and 24.2%, respectively. The differential scanning calorimetry (DSC) plots reveal that PBT polymers synthesized with I_2_ and H_2_O_2_ display an endothermic peak at 176 °C and 216 °C, respectively, probably originating from the melting of a small part of the BT oligomers [32], since they show no weight loss at the transition temperature. So, these two PBT polymers are possibly suitable for various thermoforming processing. The strongest endothermic peak at 717–718 °C with a heat flow of 0.95–1.14 mW mg^−1^ shown in the DSC plots is ascribed to the pyrolysis of residual char in the third step. The DSC behavior of PBT polymers differs significantly from that of the coordination polymer 2,5-dimercapto-1,3,4-thiadiazole-gold, which exhibits four peaks at 280, 510, 600, and 730 °C and begins to decompose at 260 °C due to the cleavage of metal-sulphur and metal-nitrogen linkages [17].

#### 3.5.3. Fluorescence Properties

**Figure 9:**
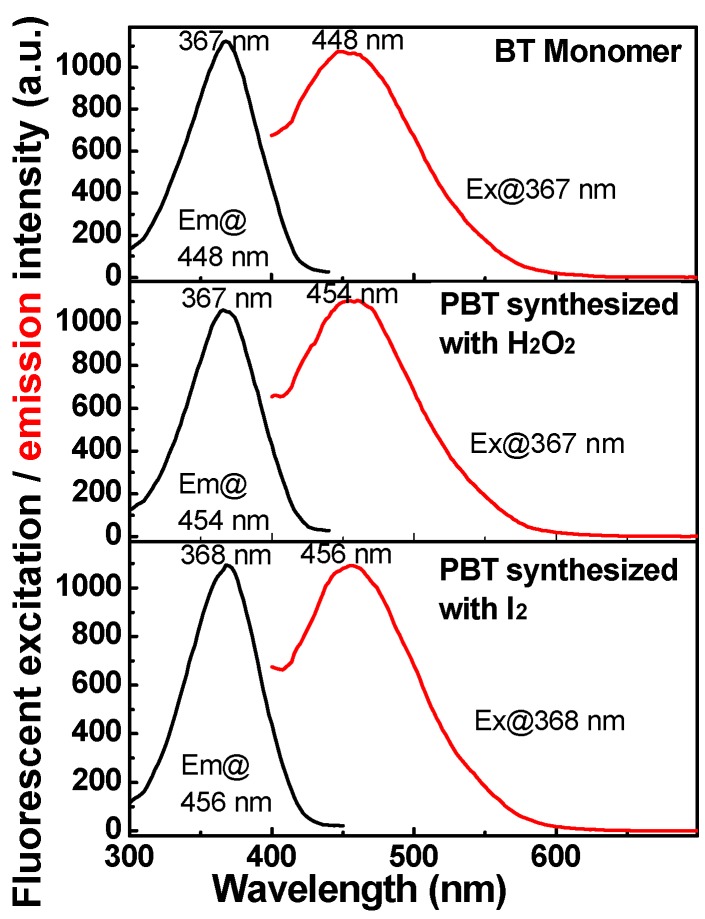
Fluorescent excitation and emission spectra obtained at fixed slit widths of 5 nm of 100 μg L^−1^ BT and PBT solutions in *N*-methy1-2-pyrrolidone (NMP).

The fluorescent excitation and emission spectra of BT monomer and PBT nanosheets synthesized with two oxidants in Figure 9 suggest that all of the PBT polymers and BT monomer exhibited analogous excitation and emission spectra, with similar shapes and peak positions. All the excitation spectra show a broad peak centered at about 367–368 nm, owning to n→π* electronic transitions found in both BT monomer and its polymers. In the case of PBT polymers, both the emission spectra generated by the radiative decay of excitons exhibit a maximal peak at 454–456 nm, suggesting that the PBT nanosheets are typical blue-light emitting materials. Note that PBT polymers emit relatively longer wavelength fluorescence than BT monomer due to the polymerization effect. Meanwhile, PBT polymer synthesized with I_2_ shows almost equivalent fluorescence emission intensity but slightly longer emission wavelength in comparison with that synthesized with H_2_O_2_. Hence, we choose PBT polymer synthesized with I_2_ for the latter fluorescence investigation.

Also, the fluorescence emission intensity of PBT solution depends markedly on its concentration (Figure S3), with the fluorescence emission culminating at 50 μg L^−1^ when excited at 368 nm, because an increased PBT concentration within a certain range would enhance the fluorophore concentration and thus the fluorescence intensity, but further increasing the PBT concentration from 50 μg L^−1^ to 200 μg L^−1^ would cause self-absorption and self-quenching effects and thereby weaken the fluorescence emission. Therefore, a PBT concentration of 50 μg L^−1^ was chosen for the subsequent fluorescence investigation.

**Figure 10:**
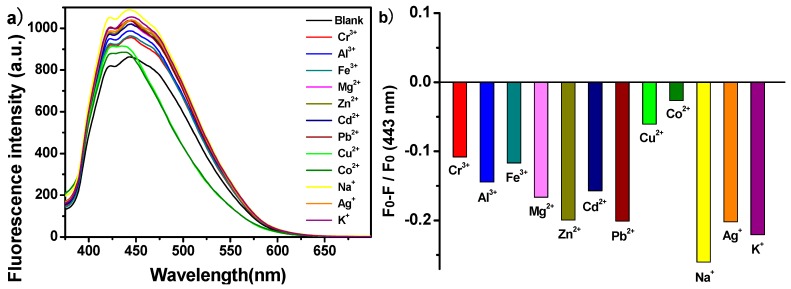
(**a**) Fluorescence spectra of PBT (50 μg L^−1^) in the absence (blank) and presence of different metal salts (1.0 × 10^−5^ mol L^−1^) recorded in an NMP/H_2_O (9:1, *v*/*v*) solvent system at pH 7.0, excitation wavelength is 350 nm; (**b**) fluorescence intensity change plot for PBT where (*F*_0_ − *F*)/*F*_0_ is plotted against each metal ion (*F*_0_ is the fluorescence intensity in the absence of metal ions, and *F* is the intensity when metal ions are present).

When maintaining the other fluorescence spectrum examination conditions unchanged except to replace the solvent pure NMP with semi-aqueous NMP/H_2_O (9:1, *v*/*v*), the optimal excitation wavelength, relative fluorescence intensity, and profiles of excitation and emission spectra underwent almost no change or insignificant change, though the emission maximum exhibited a blue shift from 456 nm to 443 nm. This signifies that the existence of a little water in the test solution may have a negligible effect on the fluorescence emission. Thus, the PBT fluorescent probe can be a candidate for the direct detection of metal ions in water. Figure 10a,b show the fluorescence emission spectra and intensity changes of the reaction mixtures of the PBT-NMP solution with different metal ions in an aqueous solution or pure water (blank). Interestingly, the fluorescence of PBT was enhanced without exception after reacting with any kind of metal ion. The fluorescence enhancement is attributed to the inhibition of the photoinduced electron transfer (PET) quenching of the excited state of the fluorophore moiety, i.e., the 1,3,4-thiadiazole ring (electron acceptor), by the lone pair electrons of S(2) or S(5) atoms (electron donor) [56,57,58]. 

#### 3.5.4. Adsorption of Heavy Metal Ions

##### Adsorption Capacity

The PBT nanosheets obtained by I_2_ and H_2_O_2_ exhibit a powerful adsorbability toward heavy metal ions because of the presence of a large amount of –S–, –SH, and =N– groups containing lone pairs of electrons on the PBT chains that are confirmed by the FT-IR and MALDI-TOF mass spectroscopies. The result of the preliminary adsorption experiment shows that when the initial Pb^2+^ concentration was up to 200 mg L^−1^ and the dosage of PBT sorbent was 1.0 g L^−1^, the adsorption capacities and adsorption ratios toward Pb^2+^ ion for PBT nanosheets obtained by I_2_ and H_2_O_2_ were 168.23 and 124.56 mg g^−1^, and 75.18% and 65.27%, respectively. It is clear that the PBT obtained with I_2_ exhibits better adsorption performance than that obtained with H_2_O_2_, so the PBT obtained with I_2_ was chosen for the further study of heavy metal ion adsorption. 

To investigate the adsorption capacity of PBT nanosheets, several heavy metal ions including Hg^2+^, Pb^2+^, Ag^+^, Co^2+^, Cr^3+^, Cd^2+^, Fe^3+^, Zn^2+^, and Cu^2+^ at the same concentrations of 200 mg L^−1^ were used for adsorption testing, and the experiments were conducted by batch test. The results are summarized in Table 2, from which one can find that the PBT nanosheets exhibited excellent adsorption capacity (*Q*) for most metal ions, with the *Q* values following the order: *Q*(Hg^2+^) > *Q*(Pb^2+^) > *Q*(Ag^+^) > *Q*(Fe^3+^) > *Q*(Cu^2+^) > *Q*(Cd^2+^) > Q(Zn^2+^) > *Q*(Co^2+^) > *Q*(Cr^3+^). The highest adsorption capacity for Hg^2+^ ion was found up to 168.23 mg g^−1^ (0.84 mmol g^−1^). The polyfunctional groups including –S–, –SH, and =N– could efficiently bind metal ions through sharing lone pairs of electrons to form chelating bonds (Scheme 1), which explains why the PBT nanosheets exhibited such good adsorption capacities. 

##### Competitive Adsorption of Metal Ions

As summarized in Table 2, the theoretical selectivity coefficients of three heavier Hg^2+^, Pb^2+^, and Ag^+^ ions versus six lighter ions range from 1.35 to 7.35, signifying that coexisting lighter ions cannot greatly influence the robust adsorbability of PBT nanosheets toward heavier Hg^2+^ and Ag^+^ ions, as shown in Table 3. The adsorption ratio (*q*, %) order onto the nanosheets was Hg^2+^ > Ag^+^ > Fe^3+^ > Cu^2+^ > Cr^3+^ > Co^2+^ > Cd^2+^ > Zn^2+^ > Pb^2+^. The most powerful adsorbability of Hg^2+^ and Ag^+^ ions onto the nanosheets was not significantly affected by the seven coexisting metal ions, while the adsorbability of Pb^2+^ was at a disadvantage in the competitive adsorption. The results of selectivity experiments imply that PBT nanosheets showed stronger affinity to Hg^2+^ and Ag^+^ ions rather than Fe^3+^, Cu^2+^, Cr^3+^, Co^2+^, Cd^2+^, Zn^2+^, and Pb^2+^ ions in competitive adsorption systems. According to the soft and hard acids and bases (SHAB) theory [59,60], Hg^2+^ and Ag^+^ are classified as soft acids, and the –S–, –SH, and =N– groups behave as soft bases. This explains why the basic PBT nanosheets preferentially adsorb the acidic metal ions such as Hg^2+^ and Ag^+^.

##### Adsorption Kinetics

**Figure 11:**
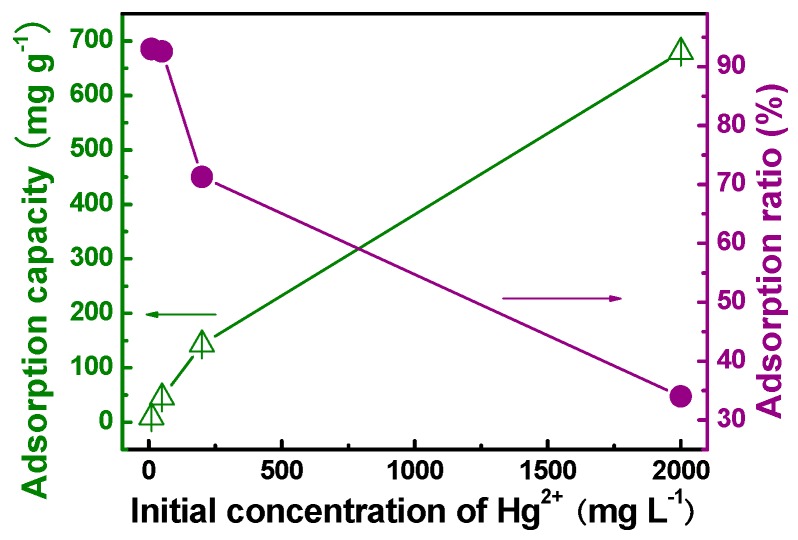
Adsorption capacity and adsorption ratio of Hg^2+^ onto 50 mg of PBT at 25 °C in 50 mL solution at different initial ion concentrations for 24 h.

The adsorption kinetics of PBT nano-sorbent was studied using Hg^2+^ as a target analyte. The effect of variation of the initial Hg^2+^ concentration on the adsorption capacity and adsorption ratio by PBT nanosheets is shown in Figure 11. The initial Hg^2+^ concentration was varied from 10 mg L^−1^ to 2000 mg L^−1^ and equilibrated for 24 h at a fixed sorbent dose of PBT. It is apparent that the adsorption capacity of Hg^2+^ ion by PBT nanosheets increased with increasing Hg^2+^ concentration, but the adsorption ratio behaved in the opposite way. At a lower concentration, Hg^2+^ ions in the aqueous solution would interact with the binding sites but the binding sites were ultimately unsaturated, thus facilitating an adsorption ratio of 93% but an adsorption capacity of only 9.30 mg g^−1^. At higher concentrations, more Hg^2+^ ions are left unadsorbed in the solution because of the saturation of the binding sites, resulting in a decreased adsorption ratio but increased adsorption capacity. This indicates that energetically less favorable sites become involved with increasing ion concentrations in an aqueous solution [61]. Notably, the highest adsorption capacity can reach up to 680.01 mg g^−1^ (3.39 mmol g^−1^), which is much higher than that for most polymer sorbents [62,63,64,65].

**Figure 12:**
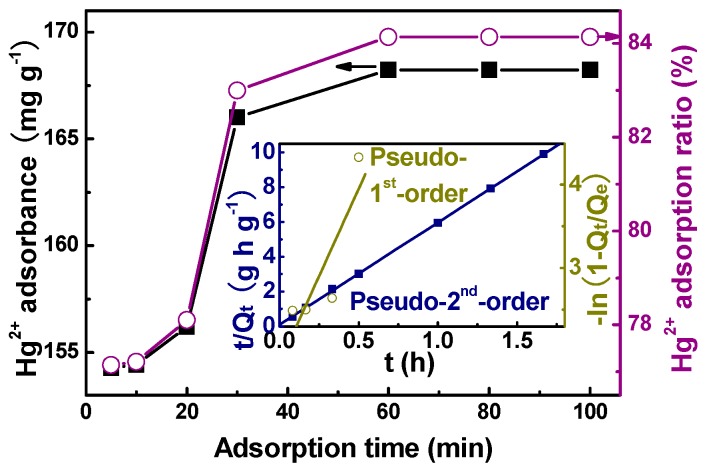
Adsorption kinetics of Hg^2+^ ions onto PBT nanosheets.

The rate at which adsorption takes place is most important when the adsorbent is employed for actual sewage treatment. Consequently, it is important to establish the contact time dependence of such an adsorbent adsorbing Hg^2+^ ions. Figure 12 shows the plot of Hg^2+^ adsorbance and adsorption ratio versus contact time onto the PBT nanosheets obtained by I_2_ to investigate the adsorption kinetics and discover the time of reaching equilibrium adsorption. The adsorption takes place very rapidly within the initial 5 min. The Hg^2+^ adsorption capacity and adsorption ratio at an adsorption time of 5 min are even as high as 154.27 mg g^−1^ and 77.14%, respectively, which are 91.7% of those at the equilibrium adsorption. However, the adsorption slows down after 5 min. It was revealed that the adsorption process comprises two steps, i.e., an initial primary rapid step and a subsequent secondary slow step [33]. Correspondingly, the rapid adsorption should take place on the nanosheet surfaces due to the immediate chelation between the Hg^2+^ ions and exposed active sites of the PBT chains. In contrast, the slow step may occur inside the nanosheets, representing the diffusion of the Hg^2+^ ions into the interior of the nanosheets. The electrostatic repulsion between the free and adsorbed Hg^2+^ ions on the surface of nanosheets makes it difficult for free and bulky mercury ions to enter into the interior of the nanosheets. 

To gain a deep insight into the adsorption kinetics of the removal of Hg^2+^ by PBT adsorbent, the experimental data were fitted into the pseudo-first-order and pseudo-second-order kinetics equations [66,67]. The curves regarding the equations of −ln(1 − *Q*_t_/*Q*_e_) = *k*_1_*t* and *t*/*Q*_t_ = *t*/*Q*_e_ + 1/*h*_0_ in the inset of Figure 12 produce relevant parameters including rate constants and correlation coefficients, as shown in Table 4. The correlation coefficient, *R*^2^, showed that the pseudo-second-order kinetic model, an indication of chemisorption mechanism, fits better with the experimental data than the pseudo-first-order kinetic model. Thus, the adsorption can be well interpreted as a chemisorption process based on the assumption of the rate determining step comprising the formation of coordination bonds between the sorbent and adsorbate, in addition to surface adsorption and intraparticle diffusion [61]. Amazingly, it was found that the adsorption time at equilibrium is even shorter, down to 1.0 h for Hg^2+^. Furthermore, the PBT adsorbents show very rapid initial adsorption rates with a rate constant *h*_0_ of up to 10,653 mg g^−1^ h^−1^ according to the pseudo-second-order kinetics. No higher *h*_0_ value than this could be found in the literature [62,68,69]. Such a rapid adsorption is due to a great number of various electron-rich groups including –S–, –SH, and =N– on the PBT nanosheets, as well as their high porosity which is confirmed by the low density (apparent density, 0.521 g cm^−3^; bulk density, 0.813 g cm^−3^) and moderate specific area (15.85 m^2^ g^−1^) in Figure S4 of the Supplementary Materials.

##### Adsorption Mechanism

The adsorption mechanism of PBT nanosheets for Hg^2+^ was further studied by XPS spectral analysis (Figure 5). Several strong peaks with BEs of 163.2, 284.7, and 398.4 eV correspond to S 2p, C 1s, and N 1s orbitals, respectively, which exist in pristine PBT nanosheets (Figure 5a). Upon adsorbing Hg^2+^ ions, a new shoulder peak with a BE of approximately 99.6–102.8 eV appears (Figure 5a,b), which confirms that Hg^2+^ ions were adsorbed onto PBT nanosheets. The high-resolution XPS spectra of C 1s, N 1s, and S 2p core levels are shown in Figure 5c–e, and the corresponding BEs are summarized in Table 1. The high-resolution C 1s XPS spectrum of PBT exhibited negligible changes after the adsorption of Hg^2+^ (Figure 5c), indicating that carbon atoms were not involved in the adsorption of mercury. By contrast, both the high-resolution XPS spectra of N 1s and S 2p for PBT presented a new group of bands with BEs of 401.3 and 161.8/163.0 eV after the adsorption of mercury, respectively, which are respectively attributed to N-Hg and S-Hg coordination bonds, not discarding the possibility of the existence of protonated nitrogen and sulfur atoms. Since the aqueous solution of the adsorbate Hg(NO_3_)_2_ is acidic, the electron-donating nitrogen and sulfur atoms are readily bound to H^+^ ions to form N–H and S–H bonds. After all, the formation of N–Hg and S–Hg coordination bonds suggests that nitrogen and sulfur atoms are the active sites of mercury ion adsorption.

Based on the XPS analysis, it is reasonable to infer that carbon atoms did not contribute to the chemical adsorption of metal ions. The adsorption mechanism could be that electron-rich –S–, –SH, and =N– functional groups in PBT nanosheets coordinated with Hg^2+^ ions to form a stable complex (Scheme 1), leading to excellent adsorbability.

##### Comparison of Hg^2+^ Adsorbability with Other Adsorbents

On the basis of a careful comparison of the Hg^2+^ adsorbability onto several typical adsorbents summarized in Table 5 [62,63,64,65,68,69,70,71,72,73,74,75], the PBT nanosheets have an even stronger adsorbability toward Hg^2+^ solutions than the other reported adsorbents, to the best of our knowledge. The adsorption ratio of magnetic functional metal-organic Fe_3_O_4_@Zn(btb) nanospheres [68] and cross-linked magnetic chitosan-phenylthiourea (CSTU) [69] at the dosage of 1.0 g L^−1^ toward 200 mg L^−1^ Hg^2+^ is only 64.93% and 67.50%, respectively. However, the PBT nanosheets at the same dosage achieved an 84.12% adsorption ratio. At the same time, PBT is an efficient adsorbent having a high adsorption capacity. when the initial concentration of mercury ions was increased to 2000 mg L^−1^, the adsorption capacity of PBT significantly rose up to 680.01 mg g^−1^, which is about two times that of polystyrene-triethylenetetramine resin (PS-TETA) [64]. The fact that the PBT nanosheets have powerful adsorbability toward a concentrated Hg^2+^ solution of 200–2000 mg L^−1^ hints that the PBT nanosheets will demonstrate very strong treating capability and wonderful durability if treating diluted Hg^2+^ solutions. Besides, the PBT nanosheets can accomplish equilibrium adsorption toward 200 mg L^−1^ Hg^2+^ solution in 1.0 h at a very fast initial adsorption rate of 10,653 mg g^−1^ h^−1^, but most of the other sorbents could reach equilibrium adsorption in 1.5–24 h at a much slower initial adsorption rate. Therefore, satisfactory Hg^2+^ adsorbability should be ascribed to an optimal synergic combination of the three kinds of active groups with moderate specific area of the PBT nanosheets.

## 4. Conclusions

Unique poly(2,5-dimercapto-1,3,4-thiadiazole) (PBT) nanosheets have been successfully synthesized through a simple chemical oxidative polymerization under environmentally friendly conditions. The as-synthesized PBT polymers possess several unique characteristics: facile one-pot synthesis; green synthesis conditions (oxidant, I_2_ or H_2_O_2_; medium, ethanol); high total molar content of sulfur and nitrogen (above 56%); high polymerization yield (up to 98.47%); and high chemoresistance (thermal and chemical stability). The polymerization media, oxidant species, monomer concentrations, oxidant/monomer molar ratios, and polymerization temperature were thoroughly optimized for the formation of uniform PBT nanosheets with a high polymerization yield, tunable size, good chemical resistance except for ammonia, high thermostability, and interesting blue-light emitting fluorescence, as well as high heavy metal ion adsorbability. The UV-Vis, FT-IR, and MALDI/TOF mass spectra, together with the solubility experiment, showed that high molecular weight PBTs were created via S-S linkage at the S(2) and S(5) positions of the monomers. The SEM and TEM analyses show that the as-prepared PBT polymers were nanosheets with a thickness of 89~367 nm. The nanoscale and sulfur/nitrogen-rich features endow this kind of polymer with wonderful performance. Most importantly, PBT nanosheets can be excellent adsorbents for mercury ions, with an adsorption capacity as high as 680.01 mg g^−1^ and a very rapid initial adsorption rate of up to 10,653 mg g^−1^ h^−1^, which makes them very competitive with most good Hg^2+^-sorbents previously reported. With these impressive functionalities, PBT nanosheets are seen as promising for many target applications, such as in water treatment, sensors, and electrodes. 

## Figures and Tables

**Table 1:** Assignment of main XPS spectral bands based on their binding energies (BEs) and atomic concentrations (ACs) for BT, PBT, and Hg^2+^-loaded PBT.

Element	BT Monomer		PBT Polymer		PBT-Hg^2+^		Assignments
	BE (eV)	AC (%)	BE (eV)	AC (%)	BE (eV)	AC (%)	
C 1s	284.8	11.6	284.8	12.0	284.9	17.5	adventitious carbon (C–C, C=C)
C 1s	286.3	15.2	286.3	18.8	286.4	27.6	C–N, C=N
C 1s	287.5	30.0	287.6	7.1	287.7	4.3	C–S, C=S
Total C	-	56.86	-	37.89	-	49.38	
N 1s	400.5	8.9	399.5	25.59	399.5	16.3	–N=, N–N
N 1s	401.3	8.8	-	-	401.3	2.9	N–H, or N–Hg
Total N	-	17.61	-	25.59	-	19.20	
S 2p	162.3	5.2	-	-	161.8	2.0	S–H, or S–Hg
S 2p	163.5	2.6	-	-	163.0	1.0	S–H, or S–Hg
S 2p	164.7	11.9	164.1	24.3	164.2	18.9	heterocyclic sulfur (C–S, C=S), or S–S
S 2p	165.8	5.9	165.3	12.1	165.4	9.4	heterocyclic sulfur (C–S, C=S), or S–S
Total S	-	25.53	-	36.43	-	31.23	
Hg 4f_7/2_	-	-	-	-	100.5	0.18	N–Hg, S–Hg

**Table 2:** Adsorption capacity and adsorption ratio of nine metal ions at an initial metal concentration of 200 mg L^−1^ and an adsorption temperature of 25 °C for a contact time of 24 h with a PBT sorbent dosage of 1.0 g L^−1^.

Metal Ion Solutions	Adsorption Capacity (mg g^−1^)	Adsorption Ratio (%)	Theoretical Selectivity Coefficient		
			Hg^2+^	Pb^2+^	Ag^+^
Hg(NO_3_)_2_	168.23	84.12	1.00	0.89	0.43
Pb(NO_3_)_2_	149.77	75.18	1.12	1.00	0.48
AgNO_3_	71.87	36.29	2.34	2.08	1.00
Co(NO_3_)_2_	28.22	14.36	5.96	5.31	2.55
Cr(NO_3_)_3_	22.89	11.47	7.35	6.54	3.14
Cd(NO_3_)_2_	42.35	21.46	3.97	3.54	1.70
Fe(NO_3_)_3_	53.27	30.12	3.16	2.81	1.35
Zn(NO_3_)_2_	36.22	18.13	4.64	4.14	1.98
Cu(NO)_2_	50.30	25.15	3.34	2.98	1.43

**Table 3:** Competitive adsorption of nine metal ions onto 50 mg of PBT at 25 °C in 50 mL solution at the same initial ion concentration of 20 mg L^−1^ for each metal ion for 24 h.

Mixed Solution	Metal Ions	Adsorption Capacity (mg g^−1^)	Adsorption Ratio (%)
Hg(NO_3_)_2_	Hg^2+^	19.22	98.38
Pb(NO_3_)_2_	Pb^2+^	10.38	53.13
AgNO_3_	Ag^+^	17.00	87.06
Co(NO_3_)_2_	Co^2+^	11.72	60.01
Cr(NO_3_)_3_	Cr^3+^	11.78	60.30
Cd(NO_3_)_2_	Cd^2+^	11.32	57.96
Fe(NO_3_)_3_	Fe^3+^	12.33	63.11
Zn(NO_3_)_2_	Zn^2+^	11.11	56.88
Cu(NO)_2_	Cu^2+^	12.11	61.99

**Table 4:** Kinetic model equations for Hg^2+^ adsorption onto PBT nanosheets.

Mathematical Model	Equation	*R*^2^	Standard Deviation	*k*_1_ (h^−1^) *k*_2_ (g mg^−1^ h^−1^) *h*_0_ = *k*_2_*Q*_e_^2^ (mg g^−1^ h^−1^)	*Q*_e_ Found by	
					Experiment	Equation
Pseudo-1st-order	−ln(1 − *Q*_t_/*Q*_e_) = 4.1913*t* + 1.8535	0.7491	0.5490	*k*_1_ = 4.1913 *k*_2_ = 0.3764 *h*_0_ = 1.0653 × 10^4^	168.23	-
Pseudo-2nd-order	*t*/*Q*_t_ = 0.00588*t* + 9.3872 × 10^−5^	0.9988	4.4622 × 10^−5^		168.23	170.07

**Table 5:** Comparison of specific surface area (S_BET_), average pore diameter, equilibrium adsorption capacity (*Q*_e_) and adsorption ratio (*q*), and initial adsorption rate (*h*_0_) of various adsorbents towards Hg^2+^.

Adsorbents	S_BET_ (m^2^ g^−1^)	Pore Diameter (nm)	[Hg^2+^]_0_ (mg L^−1^)	Adsorbent Dose (g L^−1^)	pH	*Q*_e_ (mg g^−1^)	*q* (%)	*h*_0_ (mg g^−1^ h^−1^)/*t*_Qe_ (h)
PBT (this work)	15.85	19.4	200 (2000)	1.0	4.68	168.23 (680.01)	84.12 (34)	10,653/1.0
Melamine-based porous polymer networks [62]	153	14.3	401.18	1.77	-	198	87.50	1.41/24
Fe_3_O_4_@Zn(btb) nanospheres [68]	-	-	200	1.0	8	129.87	64.93	0.059/0.5
Cross-linked magnetic chitosan-phenylthiourea (CSTU) [69]	64.5	860	200	1.0	5	135	67.50	139.8/3
Dithiocarbamate chelating resin (DTMAN) [70]	46.82	4.09	1003	1.0	5	461.35	46.00	-/0.33
Hierarchical micro-nano porous carbon material (MNC) [71]	1978	540	600	1.0	6	241.2	40.20	-/1.66
Polyvinyl alcohol (PVA) [72]	-	12	30	1.0	7	25.5	85.00	-/2
Poly(1-amino-5-chloroanthraquinone) (PACA) [63]	35	-	100.3	1.0	6	83.25	83.00	-/2
Polystyrene-triethylenetetramine resin (PS-TETA) [64]	-	-	2000	1.0	6	344.8	17.24	-/14
Ethylenediamine-modified magnetic crosslinking chitosan microspheres (EMCR) [73]	68.6	826	1003	1.0	3	539.58	53.80	-/2
Wool-grafted-poly(cyano-acetic acid α-amino-acrylichydrazide) (wool-g-PCAH) chelating fibers [74]	-	-	100	0.60	5	154.32	93.3	-/1.5
Impregnated resin containing 1-(2-thiazolylazo)-2-naphthol (TAN) [75]	750	10	1000	1.0	5.5	450.45	45.04	-/0.92
Modified poly(ethylene terephthalate) chelating fibers (PET-TSC) [65]	5.785	-	150	1.0	5	120.02	80.01	-/0.83

## Supplementary Materials

The following are available online at www.mdpi.com/2073-4360/10/1/24/s1, Table S1: Solubility and solution color of BT monomer and PBT polymers, Table S2: Main composition and proportion of frontier orbitals in BT, Table S3: Main atomic electron spin densities for BT, Table S4: FT-IR spectra data of solid BT and PBT and their assignments, Table S5: WAXD data of solid BT and PBTs, Table S6: Proposed composition and corresponding theoretical mass-to-charge ratio of PBT molecules, Figure S1: UV-Vis spectra of BT monomer and PBT polymers, Figure S2: Molecular model of BT monomer with minimized energy, Figure S3: Fluorescent emission spectra of PBT solutions in NMP at different concentrations, Figure S4: Nitrogen adsorption-desorption isotherms and pore size distribution curves of fine PBT powders.
