# Supplementary material for: Highly Productive Synthesis, Characterization, and Fluorescence and Heavy Metal Ion Adsorption Properties of Poly(2,5-dimercapto-1,3,4-thiadiazole) Nanosheets
Source: Polymers (Basel). 2017 Dec 25;10(1):24. doi: 10.3390/polym10010024 (PMC6414834; doi:10.3390/polym10010024)
Supplement: Supplementary file 1 [file polymers-10-00024-s001.pdf]

polymers

Supplementary Materials: Highly Productive Synthesis, Characterization, and Fluorescence and Heavy Metal Ion Adsorption Properties of Poly(2,5-dimercapto-1,3,4-thiadiazole) Nanosheets

Chao Li [1,2], Shaojun Huang [1,*], Chungang Min [1], Ping Du [1], Yi Xia [1], Chaofen Yang [1] and Qiuling Huang [1]

[1] Research Center for Analysis and Measurement, Kunming University of Science and Technology, Kunming 650093, China; lichao2527@yeah.net (C.L.); minchungang@163.com (C.M.); dupin515@163.com (P.D.); xiayi0125@163.com (Y.X.); yangmlh@163.com (C.Y.); hql1975@eyou.com (Q.H.)

[2] School of Materials Science and Engineering, Kunming University of Science and Technology, Kunming 650093, China

* Correspondence: huangshaojun1975@163.com or sjhuang@kmust.edu.cn; Tel.: +86-0871-6511-9674

Table S1. Solubility and solution color of BT monomer and PBT polymers prepared with (a) an I_2/BT molar ratio of 1.5 and an initial BT concentration of 50 mmol L^{-1}, and (b) an H_2O_2/BT molar ratio of 3.53 and an initial BT concentration of 58.25 mmol L^{-1} in ethanol at 25 °C for 24 h.

Solvent	Solubility and solution color of PBT polymers and BT monomer		
	PSA polymers prepared by two oxidants		BT monomer
	(a) I_2	(b) H_2O_2	
1 mol L^{-1} HCl	insoluble	insoluble	slightly soluble, pale yellow
0.1 mol L^{-1} $NH_3{\cdot}H_2O$	soluble, colorless	soluble, colorless	soluble, colorless
THF	insoluble	slightly soluble, colorless	soluble, yellow
DMF	slightly soluble, pale yellow	slightly soluble, pale yellow	soluble, dark yellow
NMP	slightly soluble, pale yellow	mainly soluble, colorless	soluble, yellowish-brown
DMSO	slightly soluble, pale yellow	partially soluble, pale yellow	soluble, pale yellow
C_2H_5OH	insoluble	slightly soluble, colorless	soluble, pale yellow
CH_3OH	insoluble	slightly soluble, colorless	soluble, pale yellow
CH_3COCH_3	insoluble	slightly soluble, colorless	soluble, colorless
CH_3NO_2	insoluble	slightly soluble, colorless	partially soluble, pale yellow

Table S2. Main composition and proportion (%) of frontier orbitals in BT.

Atom	HOMO–1	HOMO	LUMO	LUMO+1
S(1)	28.62	0.07	19.76	8.18
C(2)	0.10	8.81	28.41	39.52
S(2)	35.55	30.45	5.16	5.55
N(3)	0.04	10.71	6.54	0.48
N(4)	0.04	10.71	6.54	0.48
C(5)	0.10	8.81	28.41	39.52
S(5)	35.55	30.45	5.16	5.55

Table S3. Main atomic electron spin densities for BT.

Atom	Electron spin density	Atom	Electron spin density
S(1)	-0.041207	N(4)	0.145016
C(2)	0.055810	C(5)	0.055811
S(2)	0.330757	S(5)	0.330757
N(3)	0.145017		

Table S4. FT-IR spectra data of solid BT and PBT and their assignments [1–6].

IR data/cm^{-1} of solid BT	IR data/cm^{-1} of solid PBT synthesized with		Assignment
	(a) I_2	(b) H_2O_2	
3404 (w)	3438 (s)	3437 (s)	υ (adventitious H_2O)
3056 (w)	2977(w), 2913 (w)	2976 (w), 2915 (w)	υ (N_{ring}–H)
2476 (w)	-	-	$υ_{s.}$ (S–H)
1637 (w)	1635 (s)	1635 (s)	$υ_{s.}$ (C=N)
1501 (vs)	-	-	δ $(N–H)_{ip.}$
1447 (s)	-	-	δ $(N–H)_{rock}$
1387 (m)	1381 (vs)	1380 (vs)	υ (thiadiazole ring skeleton)
1262 (vs)	1245 (w)	1251 (w)	thioamide II mode
1119 (s)	1122 (w)	1119 (w)	υ (thiadiazole ring skeleton)
1049 (vs)	1038 (vs)	1041 (vs)	υ (N–N)
938 (m)	-	-	υ (C=S)
749 (m)	744 (w)	744 (w)	δ $(N–H)_{tor.}$
712 (vs)	713 (w)	715 (w)	$υ_{as.}$ (C–S–C endocyclic)
653 (m)	646 (w)	645 (w)	$υ_{s.}$ (C–S–C endocyclic)
533 (w)	529 (w)	531 (w)	υ (S–C–S)
-	492 (w)	492 (w)	υ (S–S)

w: weak, m: medium, s: strong, vs: very strong, υ: stretch, δ: deformation, as.: asymmetric, s.: symmetric, ip.: in-plane, tor.: torsion

Table S5. WAXD data of solid BT and PBTs synthesized with I_2 and H_2O_2.

Analyte	Bragg angle 2θ
BT monomer	13.3, 15.5, 17.0, 17.6, 18.1, 19.3, 19.8, 20.9, 22.1, 22.5, 22.9, 23.5, 25.5, 25.8, 26.5, 26.9, 27.8, 29.4, 29.8, 30.2, 30.9, 31.3, 32.1, 32.7, 33.0, 33.4, 33.7, 35.5, 37.2, 37.5, 38.1, 38.6, 39.8, 40.3, 40.9, 41.5, 42.4, 43.5, 45.2, 46.1, 48.7, 51.1, 52.5, 53.1
PBT synthesized with I_2	17.7, 17.9, 18.6, 20.6, 20.8, 26.2, 27.8, 31.9, 32.6, 33.0, 33.9, 35.8, 36.3, 36.9, 37.5, 37.9, 38.8, 41.1, 42.9, 43.2, 45.3, 48.9, 49.8, 51.9, 53.3, 57.4
PBT synthesized with H_2O_2	17.8, 18.0, 18.7, 20.7, 20.9, 26.3, 27.9, 32.0, 32.7, 33.1, 34.0, 35.9, 36.4, 37.0, 37.6, 38.0, 38.9, 41.1, 43.0, 43.4, 45.4, 49.0, 49.9, 52.0, 53.4, 57.5

Table S6. Proposed composition and corresponding theoretical mass-to-charge ratio of PBT molecules synthesized with I_2 and H_2O_2.

PBT synthesized with I_2			PBT synthesized with H_2O_2		
Experimental value of *m*/*z*	Proposed composition	Calculated value of *m*/*z*	Experimental value of *m*/*z*	Proposed composition	Calculated value of *m*/*z*
859.1	$[H(C_2N_2S_3)_6H{-}S]^+$	859.2	863.1	$[H(C_2N_2S_3)_6H{-}S+4H]^+$	863.2
968.1	$[H(C_2N_2S_3)_6H{-}S+Ag+H]^+$	968.1	897.0	$[H(C_2N_2S_3)_6H{-}S+K]^+$	898.3
1001.6	$[H(C_2N_2S_3)_6H+Ag+2H]^+$	1001.2	971.0	$[H(C_2N_2S_3)_6H+2K+H]^+$	970.5
1085.1	$[H(C_2N_2S_3)_7H+2Na+2H]^+$	1085.5	1004.7	$[H(C_2N_2S_3)_6H+Ag+5H]^+$	1004.2
1185.8	$[H(C_2N_2S_3)_8H]^+$	1187.7	1085.9	$[H(C_2N_2S_3)_7H+2Na+2H]^+$	1085.5
1300.7	$[H(C_2N_2S_3)_8H+Ag+5H]^+$	1300.6	1120.0	$[H(C_2N_2S_3)_8H{-}2S]^+$	1123.7
1401.2	$[H(C_2N_2S_3)_8H+2Ag]^+$	1403.5	1186.4	$[H(C_2N_2S_3)_8H]^+$	1187.7
1514.4	$[H(C_2N_2S_3)_8H+3Ag+3H]^+$	1514.4	1228.5	$[H(C_2N_2S_3)_8H+K+2H]^+$	1228.8
1618.1	$[H(C_2N_2S_3)_8H+4Ag]^+$	1619.3	1301.8	$[H(C_2N_2S_3)_9H{-}S]^+$	1303.8
1728.7	$[H(C_2N_2S_3)_8H+5Ag+H]^+$	1728.2	1343.9	$[H(C_2N_2S_3)_9H{-}S+K+H]^+$	1343.9
1834.0	$[H(C_2N_2S_3)_8H+6Ag]^+$	1835.1	1402.9	$[H(C_2N_2S_3)_8H+2Ag]^+$	1403.5
1942.0	$[H(C_2N_2S_3)_8H+7Ag]^+$	1943.0	1452.4	$[H(C_2N_2S_3)_{10}H{-}S]^+$	1452.1
2050.2	$[H(C_2N_2S_3)_8H+8Ag]^+$	2050.9	1524.8	$[H(C_2N_2S_3)_{10}H+K+H]^+$	1524.1
2265.5	$[H(C_2N_2S_3)_{15}H+K+H]^+$	2265.3	1567.6	$[H(C_2N_2S_3)_{10}H++K+2Na]^+$	1569.2
2377.2	$[H(C_2N_2S_3)_{16}H+3H]^+$	2376.4	1619.5	$[H(C_2N_2S_3)_8H+4Ag]^+$	1619.3
2481.1	$[H(C_2N_2S_3)_{16}H+Ag]^+$	2481.3	1676.3	$[H(C_2N_2S_3)_{11}H+2Na]^+$	1678.3
2591.5	$[H(C_2N_2S_3)_{16}H+2Ag+2H]^+$	2591.2	1728.6	$[H(C_2N_2S_3)_8H+5Ag+H]^+$	1728.2
2696.5	$[H(C_2N_2S_3)_{16}H+3Ag]^+$	2697.1	1834.2	$[H(C_2N_2S_3)_8H+6Ag]^+$	1835.1
2805.1	$[H(C_2N_2S_3)_{16}H+4Ag]^+$	2805.0	1944.7	$[H(C_2N_2S_3)_8H+7Ag+H]^+$	1944.0
2910.4	$[H(C_2N_2S_3)_{16}H+5Ag]^+$	2912.9	2049.6	$[H(C_2N_2S_3)_8H+8Ag]^+$	2050.9
			2159.3	$[H(C_2N_2S_3)_{14}H+K+2Na]^+$	2162.1
			2265.0	$[H(C_2N_2S_3)_{15}H+K]^+$	2264.3
			2379.8	$[H(C_2N_2S_3)_{16}H+6H]^+$	2379.4
			2485.2	$[H(C_2N_2S_3)_{16}H+Ag+4H]^+$	2485.3
			2596.2	$[H(C_2N_2S_3)_{16}H+2Ag+7H]^+$	2596.2
			2703.3	$[H(C_2N_2S_3)_{16}H+3Ag+6H]^+$	2703.1
			2811.3	$[H(C_2N_2S_3)_{16}H+4Ag+6H]^+$	2811.0
			2923.6	$[H(C_2N_2S_3)_{19}H+Ag]^+$	2925.9
			3022.8	$[H(C_2N_2S_3)_{16}H+6Ag+2H]^+$	3022.8
			3132.8	$[H(C_2N_2S_3)_{16}H+7Ag+4H]^+$	3132.7
			3245.6	$[H(C_2N_2S_3)_{21}H+Ag+Na]^+$	3245.3
			3351.7	$[H(C_2N_2S_3)_{21}H+2Ag+Na]^+$	3353.2
			3457.6	$[H(C_2N_2S_3)_{23}H+2Na]^+$	3456.8
			3564.0	$[H(C_2N_2S_3)_{23}H+Ag+2Na]^+$	3564.7
			3670.7	$[H(C_2N_2S_3)_{24}H+Ag+3H]^+$	3670.0
			3780.6	$[H(C_2N_2S_3)_{24}H+2Ag+6H]^+$	3780.9

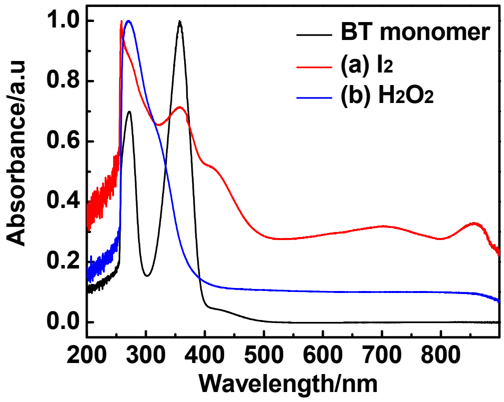

Figure S1. UV-Vis spectra of BT monomer and PBT polymers prepared with (a) an I_2/BT molar ratio of 1.5 and an initial BT concentration of 50 mmol L^{-1}, and (b) an H_2O_2/BT molar ratio of 3.53 and an initial BT concentration of 58.25 mmol L^{-1} in ethanol at 25 °C for 24 h.

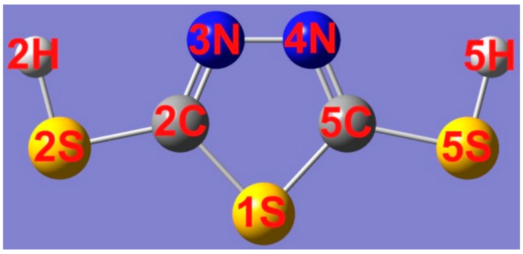

Figure S2. Molecular model of BT monomer with minimized energy.

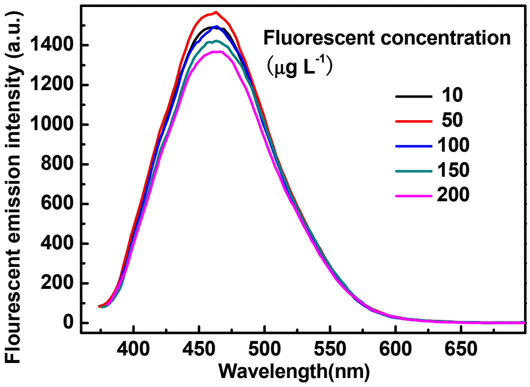

Figure S3. Fluorescent emission spectra of PBT solutions in NMP at different concentrations. The PBT was synthesized with I_2 as the oxidant.

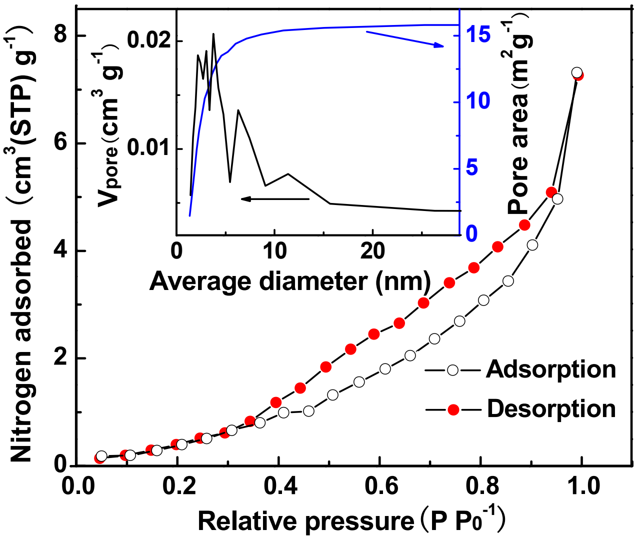

Figure S4. Nitrogen adsorption-desorption isotherms and pore size distribution curves (inset) of fine PBT powders synthesized with I_2 as oxidant.

References

1. Pope, J.M.; Sato, T.; Shoji, E.; Oyama, N.; White, K.C.; Buttry, D.A. Organosulfur/conducting polymer composite cathodes II. Spectroscopic determination of the protonation and oxidation states of 2,5-dimercapto-1,3,4-thiadiazole. *J. Electrochem. Soc.* **2002**, 149, A939–A952.
2. Wang, D.; Li, S.; Ying, Y.; Wang, M.J.; Xiao, H.M.; Chen, Z.X. Theoretical and experimental studies of structure and inhibition efficiency of imidazoline derivatives. *Corros. Sci.* **1999**, *41*, 1911–1919.
3. Shouji, E.; Oyama, N. Examination of the cleavage and formation of the disulfide bond in poly[dithio-2,5-(1,3,4-thiadiazole)] by redox reaction. *J. Electroanal. Chem.* **1996**, *410*, 229–234.
4. Zhao, Y.X.; Sun, X.Y. *Spectrometric Identification of Organic Molecular Structures*, 1st ed.; Science Press: Beijing, China, 2003; pp. 373–388, ISBN 7-03-010866-3.
5. Aydogdu, G.; Gunendi, G.; Zeybek, D.K.; Zeybek, B.; Pekyardimci, S. A novel electrochemical DNA biosensor based on poly-(5-amino-2-mercapto-1,3,4-thiadiazole) modified glassy carbon electrode for the determination of nitrofurantoin. *Sens. Actuators B Chem.* **2014**, *197*, 211–219.
6. Revin, S.B.; John, S.A. Electropolymerization of 3-amino-5-mercapto-1,2,4-triazole on glassy carbon electrode and its electrocatalytic activity towards uric acid. *Electrochim. Acta* **2011**, *56*, 8934–8940.
